# FGF receptor 2 signaling in granulosa cells is required for normal female fertility in mice

**DOI:** 10.1530/REP-25-0219

**Published:** 2025-10-28

**Authors:** Takuya Kanke, Ayaka Ichikawa, Yuki Akimoto, Shunichi Matsunaga, Wataru Fujii, Tsutomu Endo, Kunihiko Naito, Koji Sugiura

**Affiliations:** ^1^ Department of Animal Resource Sciences, Graduate School of Agricultural and Life Sciences, The University of Tokyo, Tokyo, Japan; ^2^ Department of Veterinary Medical Sciences, Graduate School of Agricultural and Life Sciences, The University of Tokyo, Tokyo, Japan

**Keywords:** ovary, fibroblast growth factor, granulosa cells, glycolysis

## Abstract

**In brief:**

Fibroblast growth factor (FGF) signaling has been implicated in ovarian follicular development in mammals, but its *in vivo* role in female fertility remains unclear. This study reveals the requirement of fibroblast growth factor receptor 2 (FGFR2) signaling in ovarian granulosa cells for normal female fertility in mice, likely by regulating glycolysis in cumulus cells and enhancing oocyte developmental competence.

**Abstract:**

FGF signaling has been implicated in ovarian follicular development in mammals, but its *in vivo* role in female fertility remains unclear. To investigate this role, we generated granulosa cell-specific conditional knockout (cKO) mice lacking FGF receptor 2 (FGFR2). *Fgfr2* cKO female mice exhibited significantly reduced fertility despite normal follicular development, estrous cycles, and ovulation. In contrast, cumulus cells in cKO mice showed decreased expression of glycolytic enzymes such as PFKP and LDHA, potentially impairing metabolic support for oocytes. Consequently, cKO cumulus cells were less competent than control cells in supporting the meiotic resumption of oocytes under *in vitro* conditions. Furthermore, while cKO oocytes developed into blastocysts at normal rates *in vitro*, their ability to reach term after embryo transfer was significantly diminished. These findings demonstrate that FGFR2-mediated FGF signaling in granulosa cells is essential for normal female fertility, likely by regulating glycolysis in cumulus cells and promoting oocyte developmental competence.

## Introduction

The development of ovarian follicles involves multiple intra- and extrafollicular factors in mammals. For example, members of the transforming growth factor β (TGF-β) superfamily, such as bone morphogenetic protein 15 (BMP15) and growth differentiation factor 9 (GDF9), are produced within follicles and play pivotal roles in female fertility. In fact, naturally occurring point mutations in the *BMP15* gene, the *GDF9* gene, or both profoundly affect fertility in ewes (Galloway *et al.* 2000, Hanrahan *et al.* 2004); likewise, female mice deficient in *Bmp15* and/or *Gdf9* exhibit a severe reduction in fertility, which is associated with impaired development of follicular somatic granulosa cells (Dong *et al.* 1996, Yan *et al.* 2001, Su *et al.* 2008). The infertility observed in these mutants highlights the essential role of BMP15/GDF9 signaling for normal follicular development and female fertility in mammals.

In addition to the TGF-β superfamily members, many fibroblast growth factor (FGF) ligands are produced within follicles, and their functions have been actively studied using *in vitro* models. For example, in bovine ovaries, FGF2 is produced by granulosa cells and has been shown to regulate granulosa cell steroidogenesis and enhance oocyte developmental competence (Neufeld *et al.* 1987, Vernon & Spicer 1994, Barros *et al.* 2019). In addition, oocyte-derived FGF8, in coordination with BMPs, regulates steroidogenesis in rat granulosa cells (Miyoshi *et al.* 2010), and in mice, oocyte-derived FGF8 together with BMP15 promote glycolysis in cumulus cells, a subpopulation of granulosa cells located in close proximity to the oocytes (Sugiura *et al.* 2005, 2007). While these studies support the idea that FGF signaling is essential for the proper regulation of ovarian function, the role of FGF signaling in mammalian female fertility is ill-defined due to the lack of mutant models deficient in ovarian FGF signals.

In mammals, the FGF family is composed of 22 ligands and 4 FGF receptors (FGFRs). As mentioned above, many FGF ligands are produced within the ovaries and are likely to act redundantly in the regulation of ovarian functions. This redundancy in FGF ligands may hinder the assessment of the ovarian requirement of FGF ligands under *in vivo* conditions using mutant mouse models. In contrast, several mouse models carry mutations in the *Fgfr* genes that exhibit altered fertility phenotypes. For example, female mice carrying an activating mutation in *Fgfr2* or *Fgfr3* are sterile (Amsterdam *et al.* 2001, Chen *et al.* 2003). Similarly, female mice with a causative mutation analogous to the human achondroplasia mutation in *Fgfr3* exhibit dwarfism and infertility (Wang *et al.* 1999). In addition, double-knockout mice for *Fgfr3* and *Fgfr4* display pronounced dwarfism and are largely infertile (Weinstein *et al.* 1998). Therefore, appropriate regulation and balance of FGFR signaling appear to be critical for normal female fertility in mice. However, since these mice also exhibit many other abnormalities, including dwarfism, the fertility defects could be attributed to general health deterioration rather than ovarian defects.

To assess the requirement for FGF signaling in ovarian function and female fertility, a mouse model with a conditional knockout (cKO) of *Fgfr2* in ovarian granulosa cells was generated using Cre recombinase driven by the *Amhr2* promoter. Using this mouse model, we present the first direct evidence that FGF signaling in granulosa cells is necessary for female fertility, at least in part by promoting oocyte developmental competence in mice.

## Materials and methods

### Granulosa cell-specific *Fgfr2* conditional knockout mice

Mice carrying an *Amhr2^Cre^* knock-in (*Amhr2^tm3(cre)Bhr^*) allele (Jamin *et al.* 2002) were provided by Dr Richard Behringer (University of Texas M.D. Anderson Cancer Center, Houston, TX, USA). The *Amhr2^Cre^* mice were mated with mice carrying a ‘floxed’ *Fgfr2* gene in which exons 8–10 were flanked by loxP sites (*Fgfr2 ^tm1Dor^/J*; The Jackson Laboratory, USA) (Yu *et al.* 2003) to generate *Fgfr2 ^flox/flox^* and *Amhr2 ^Cre/+^* mice (*Fgfr2* cKO mice). *Fgfr2* cKO mice were maintained on a C57BL/6N; CD-1 mixed background. Littermate *Fgfr2 ^flox/flox^* female mice were used as controls unless otherwise noted.

To identify these alleles, DNA was extracted from tail biopsies using the HotSHOT method (Truett *et al.* 2000) and used for PCR. The PCR primers used to detect the *Fgfr2* alleles were reported previously (Yu *et al.* 2003). The PCR primers used to detect the *Cre* allele were 5′-GCC​TGC​ATT​ACC​GGT​CGA​TGC-3′ and 5′-CAG​GGT​GTT​ATA​AGC​AAT​CCC-3′. All experiments were approved by the Animal Care and Use Committee of the University of Tokyo.

### Isolation of cumulus–oocyte complexes (COCs), cumulus cells, and mural granulosa cells

COCs and mural granulosa cells were collected from tertiary follicles of 3-week-old mice primed with 5 IU pregnant mare serum gonadotropin (PMSG, ASKA Pharmaceutical Co., Ltd, Japan) 44–48 h prior. The basic culture medium was MEMα (Thermo Fisher Scientific, USA) supplemented with penicillin G potassium (75 μg/mL, Meiji Seika Pharma, Japan), streptomycin sulfate (50 μg/mL, Meiji Seika Pharma), and bovine serum albumin (BSA, 3 mg/mL, Sigma-Aldrich, USA). During COC collection, the basic medium was supplemented with milrinone (10 μM, Sigma-Aldrich), a phosphodiesterase inhibitor, to maintain oocytes at the germinal vesicle stage. Cumulus cells were collected by stripping them from the COCs using a fine-tipped pipette with a diameter slightly smaller than that of the oocytes.

### Total RNA extraction, reverse transcription, and real-time PCR

Total RNA was extracted from cumulus cells using the ReliaPrep RNA Cell Miniprep System (Promega, USA) and reverse-transcribed using ReverTra Ace qPCR RT Master Mix with the gDNA Remover kit (TOYOBO, Japan). Real-time PCR was conducted using the THUNDERBIRD qPCR Mix (TOYOBO) and an ABI StepOnePlus real-time PCR system (Applied Biosystems, USA), according to the manufacturer’s instructions. The PCR primers used are listed in Table 1. Transcript levels were normalized to those of a housekeeping gene, *Rpl19* (ribosomal protein L19), using the 2^−ΔΔ^ method (Livak & Schmittgen 2001). The reactions were run in duplicate. Melting curve analysis was conducted at the end of the reaction to avoid false-positive signals. In addition, the PCR products were checked for correct size by agarose gel electrophoresis.

**Table 1:** Primer sets used for PCR.

Gene	RefSeq Acc. No.	Forward primer sequence (5′-3′)	Reverse primer sequence (3′-5′)
*Fgfr1*	NM_010206	AAG​CCT​GAC​CAC​CGA​ATT​GG	GAT​GCA​GGT​GTA​GTT​GCC​CT
*Fgfr2*	NM_010207	GCCAGACTTCAGCAGCCA	GTT​CAT​GGA​GGA​GCT​GGA​CT
*Fgfr4*	NM_008011	ACC​GTG​GCT​GTG​AAG​ATG​CT	TGT​GTC​TTC​CGA​TTA​GCT​TCA​TCA
*Has2*	NM_008216	CGA​GTC​TAT​GAG​CAG​GAG​CTG	GTG​ATT​CCG​AGG​AGG​AGA​GAC​A
*Ptgs2*	NM_011198	CCC​TTC​CTC​CCG​TAG​CAG​AT	TGA​ACT​CTC​TCC​GTA​GAA​GAA​CCT​TT
*Ptx3*	NM_008987	TTG​CTG​AGA​CCT​CGG​ATG​AC	GCG​AGT​TCT​CCA​GCA​TGA​TGA
*Tnfaip6*	NM_009398	ATA​CAA​GCT​CAC​CTA​CGC​CGA​A	ATC​CAT​CCA​GCA​GCA​CAG​ACA​T
*Pfkp*	NM_019703	GCC​GTG​AAA​CTC​CGA​GGA​A	GTT​GCT​CTT​GAC​AAT​CTT​CTC​ATC​AG
*Ldha*	NM_010699	TGT​GGC​AGA​CTT​GGC​TGA​GA	CTG​AGG​AAG​ACA​TCC​TCA​TTG​ATT​C
*Rpl19*	NM_009078	CCG​CTG​CGG​GAA​AAA​GAA​G	CAG​CCC​ATC​CTT​GAT​CAG​CTT
*Slc2a1*	NM_011400	GGA​ATC​GTC​GTT​GGC​ATC​CT	CAG​AGG​CCA​CAA​GTC​TGC​AT

### Immunohistochemical staining of FGFR2

The immunohistochemical detection of FGFR2 in the ovaries of *Fgfr2* cKO and control mice was performed as previously described (Maruyama *et al.* 2023). The primary antibody used was anti-FGFR2 (Bek) antibody (1:500) (sc-122, Santa Cruz Biotechnology, USA), and the signals were detected using the ImmPRESS-HRP detection kit (MP-7401, Vector Laboratories, USA) and the DAB substrate kit (SK-4105, Vector Laboratories), according to the manufacturer’s protocols.

### Mating test and evaluation of the estrous cycle

To assess the fertility of *Fgfr2* cKO mice, 2-month-old *Fgfr2* cKO or control female mice were housed with age-matched wild-type CD-1 male mice. Litter size was recorded over a 6-month period. The stages of the estrous cycle were determined by daily vaginal smears collected over 3 weeks in both *Fgfr2* cKO and control mice, following a published method (Cora *et al.* 2015).

### Histological assessment of the ovaries

Ovaries from 3-week-old or 4-month-old mutant and littermate control female mice were fixed in Bouin solution, embedded in paraffin (Pathoprep568; FUJIFILM Wako Pure Chemical Corporation, Japan), sectioned (6 μm thick), and stained with hematoxylin–eosin. Follicle counts were performed on every third section containing a visible germinal vesicle (Sugiura *et al.* 2010). Follicles were classified using the following criteria: primordial, with a single layer of flattened pregranulosa cells surrounding the oocyte; primary, with one layer of cuboidal granulosa cells; secondary, with two or more layers of granulosa cells and a theca layer; and tertiary, with a fluid-filled follicular antrum.

### Induction of cumulus expansion *in vitro*

Cumulus expansion was induced with recombinant epidermal growth factor (EGF) (10 ng/mL, PeproTech, USA) *in vitro*, as reported previously (Kanke *et al.* 2022). The degree of expansion was evaluated using the cumulus expansion index (Eppig *et al.* 1993) 14 h after EGF stimulation. The levels of transcripts encoding proteins essential for this process (HAS2, PTGS2, PTX3, and TNFAIP6) (Davis *et al.* 1999, Varani *et al.* 2002, Fulop *et al.* 2003, Sugiura *et al.* 2009) were assessed after 4 h of EGF stimulation.

### *In vitro* maturation of oocytes

To assess the competence of cumulus cells to support oocyte maturation *in vitro*, COCs from *Fgfr2* cKO mice were subjected to *in vitro* maturation. COCs were cultured in MEM (Thermo Fisher Scientific) with or without sodium pyruvate (25 μg/mL), supplemented with penicillin G potassium (75 μg/mL), streptomycin sulfate (50 μg/mL), BSA (3 mg/mL), and EGF (10 ng/mL). After 14 h of maturation culture, cumulus cells were removed from the COCs and the denuded oocytes were assessed for extrusion of the first polar body.

### *In vitro* fertilization (IVF) and embryo transfer

IVF was conducted to assess the competence of *Fgfr2* cKO mouse oocytes to support preimplantation development. Three-week-old female mice were injected with 5 IU of PMSG, followed 44–46 h later by 5 IU of human chorionic gonadotropin (hCG, Asuka Pharmaceutical, Japan) to induce ovulation. After 14 h of hCG administration, COCs were collected from the oviductal ampulla and placed in a 200 μL drop of TYH medium (Toyoda *et al.* 1971). The COCs were then inseminated with 20,000 capacitated sperm collected from wild-type CD-1 males for 6 h. The following day, the embryos were transferred from TYH medium to KSOMAA medium (Ho *et al.* 1995) and cultured for an additional 4 days. All cultures were maintained in an incubator at 37°C, 5% CO_2_, and 100% humidity.

To assess the competence of *Fgfr2* cKO oocytes to develop to term, some of the 2-cell embryos were cryopreserved using CARD methods (Nakao *et al.* 1997) until they were transferred into the oviducts of pseudopregnant CD-1 female mice at 0.5 days post coitum. Ten to twelve embryos were transferred to each recipient.

### Detection of recombined *Fgfr2* allele in blastocyst-stage embryos

To examine whether Cre-mediated recombination occurred in the oocytes of *Fgfr2* cKO mice, oocytes from either *Fgfr2* cKO or control mice were fertilized *in vitro* with sperm from wild-type males, as described above. Genomic DNA was extracted from blastocysts after 96 h of culture, as previously reported (Ikeda *et al.* 2019), and subjected to PCR to detect the floxed and deleted alleles of *Fgfr2*. Each DNA sample was prepared using two blastocysts of each genotype. PCR products were separated by agarose gel electrophoresis and visualized with ethidium bromide. The PCR primers used to detect these alleles were described previously (Yu *et al.* 2003).

### Statistical analysis

Statistical analyses were performed using JMP Pro Version 18 (SAS Institute, USA). Student’s *t*-test and the Tukey–Kramer test were used for pairwise and multiple comparisons, respectively. Percentage data were arcsine-transformed before analysis. A *P*-value of less than 0.05 was considered statistically significant. All values are presented as mean ± SEM.

## Results

### Production of granulosa cell-specific *Fgfr2* cKO mice

We generated a granulosa cell-specific cKO of *Fgfr2* in the ovary by crossing a mouse strain carrying the conditional allele of *Fgfr2* (*Fgfr2^flox^*) (Yu *et al.* 2003) with the *Amhr2^cre^* knock-in mouse strain (Jamin *et al.* 2002). The conditional allele contains loxP sites flanking exons 8–10 of *Fgfr2*, which encode alternatively spliced Ig domains IIIb and IIIc, as well as the transmembrane domain (Yu *et al.* 2003) (see discussion for details). Therefore, the protein encoded by the recombined *Fgfr2^flox^* allele, if any, would be nonfunctional due to the absence of exons required for ligand binding (IIIb or IIIc) and membrane insertion (TM domain) (Yu *et al.* 2003). This allele has been used to inactivate FGFR2 signaling in several tissues and cell types, including developing skeletal tissue (Yu *et al.* 2003), lung epithelium (Abler *et al.* 2009), and motoneurons (Fox *et al.* 2007).

**Figure 1:**
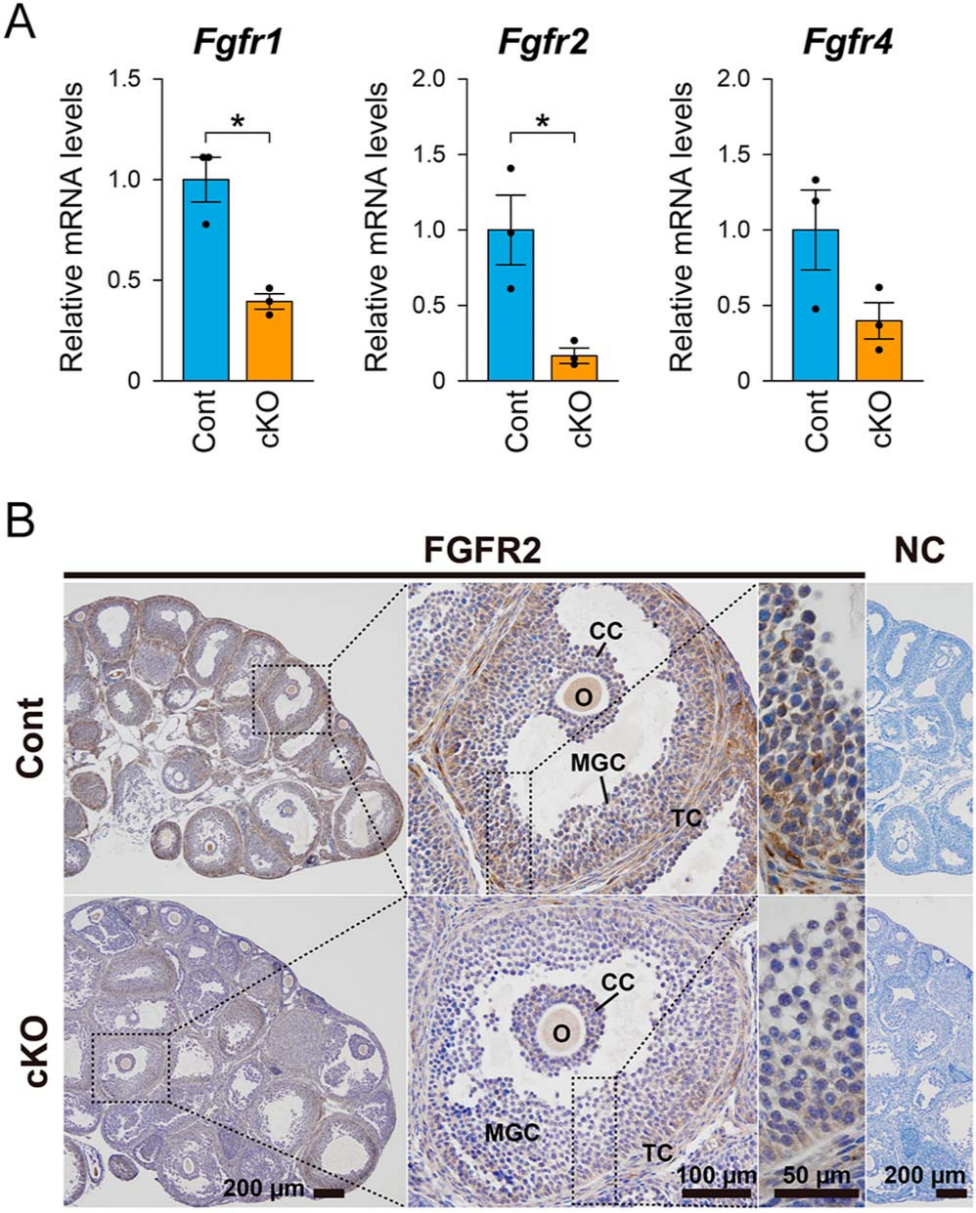
Analysis of *Fgfr* expression in cumulus cells and immunohistochemical detection of FGFR2 in ovaries of *Fgfr2* conditional knockout (cKO) and littermate control (Cont) mice. (A) Relative mRNA expression levels of *Fgfr1*, *Fgfr2*, and *Fgfr4* in cumulus cells from 3-week-old eCG-primed *Fgfr2* cKO and control mice were quantified by real-time PCR (*n* = 3). *, *P* < 0.05 (Student’s *t*-test). (B) Immunohistochemical staining was used to assess FGFR2 localization in ovaries from 3-week-old eCG-primed *Fgfr2* cKO and control mice. NC, negative control (no primary antibody); CC, cumulus cells; MGC, mural granulosa cells; O, oocytes; TC, theca cells.

A significant reduction in *Fgfr2* mRNA that occurs in granulosa cells of the cKO (*Fgfr2^flox/flox^*; *Amhr2^cre/+^*) mice was observed compared with littermate controls (*Fgfr2^flox/flox^*) (Fig. 1A). Interestingly, *Fgfr1* mRNA levels were also significantly reduced, and *Fgfr4* mRNA showed a decreasing trend in the granulosa cells of the cKO mice. *Fgfr3* transcripts were not detected under the present conditions. Therefore, signaling through FGFR2 and other FGFRs, particularly FGFR1, is likely reduced in the cKO mice.

To further confirm the reduction in FGFR2 expression in cKO mice, immunohistochemical staining was performed. Positive staining for FGFR2 was detected in granulosa cells in the control ovaries, with the strongest staining in the mural granulosa cells located at the outermost part of the follicles, gradually decreasing in cumulus cells (Fig. 1B). This staining pattern is consistent with previous findings showing that oocytes suppress *Fgfr2* mRNA expression in cumulus cells *in vitro* (Furukawa *et al.* 2014). Positive signals were also detected in oocytes and the theca layer of the control mice. The staining in granulosa cells of cKO mice was markedly reduced compared with that in control mice.

### Fertility of *Fgfr2* cKO female mice

**Figure 2:**
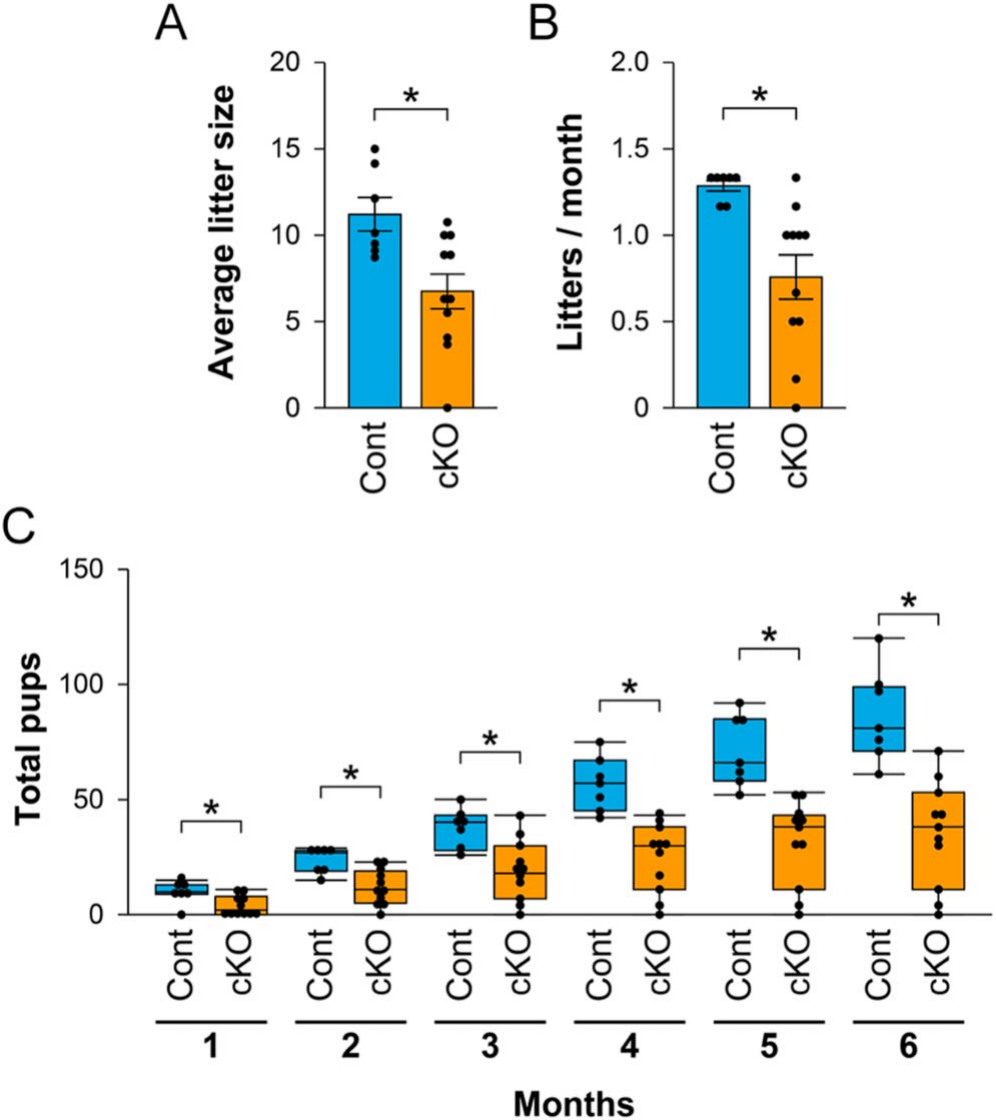
Fertility analysis in control (Cont) and *Fgfr2* cKO (cKO) female mice. (A) Average number of pups per litter and (B) number of litters per month produced by control (*n* = 7) and *Fgfr2* cKO (*n* = 11) females during a 6-month mating period. *P < 0.05 (Student’s *t*-test). (C) Total number of pups delivered per female by control (*n* = 7) and *Fgfr2* cKO (*n* = 11) mice during the 6-month period. *P < 0.05 (Student’s *t*-test).

To test the fertility of *Fgfr2* cKO female mice, 8-week-old *Fgfr2* cKO and control mice were mated with wild-type males for 6 months, and the numbers of pups and litters per month were recorded. The *Fgfr2* cKO females exhibited significantly smaller litters (6.7 ± 1.0 pups/litter) and lower litter frequency (0.8 ± 0.1 litters/month) compared with controls (11.2 ± 1.0 pups/litter and 1.3 ± 0.03 litters/month, respectively) (*P* < 0.05) (Fig. 2A and B). In addition, the cumulative number of pups produced was significantly reduced in *Fgfr2* cKOs, with only 35.2 ± 6.7 pups per female born at the end of the 6-month breeding period compared with controls (86.4 ± 7.6 pups/female) (Fig. 2C). Therefore, granulosa cell-specific *Fgfr2* cKO female mice are subfertile.

### Morphology of the *Fgfr2* cKO ovary

**Figure 3:**
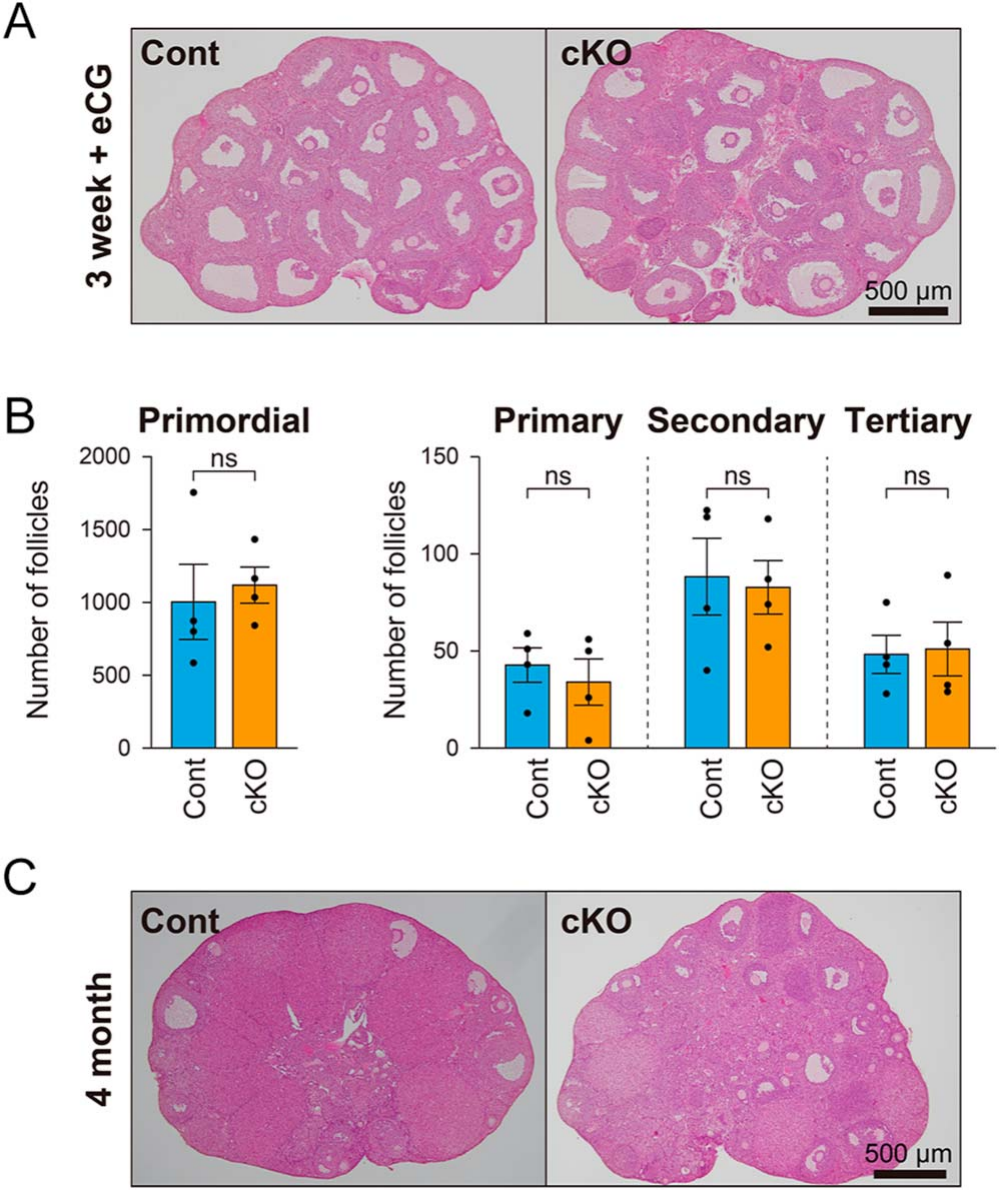
Morphology of *Fgfr2* cKO ovaries. Representative photographs of ovaries from (A) 3-week-old eCG-primed and (C) 4-month-old *Fgfr2* cKO and control mice. Scale bar = 500 μm. (B) Follicle counts in ovaries from 3-week-old eCG-primed *Fgfr2* cKO and control mice. No statistically significant differences were observed in follicle numbers at any developmental stage between groups. ns, not significant (*P* > 0.05, Student’s *t*-test).

The ovaries of 3-week-old and 4-month-old *Fgfr2* cKO mice exhibited normal histological appearance compared with control mice (Fig. 3A and C). No significant differences were observed in the number of primordial, primary, secondary, or tertiary follicles between 3-week-old *Fgfr2* cKO and control ovaries (Fig. 3B). Therefore, follicular development in *Fgfr2* cKO ovaries appears morphologically normal.

### Estrous cycle in *Fgfr2* cKO mice

**Figure 4:**
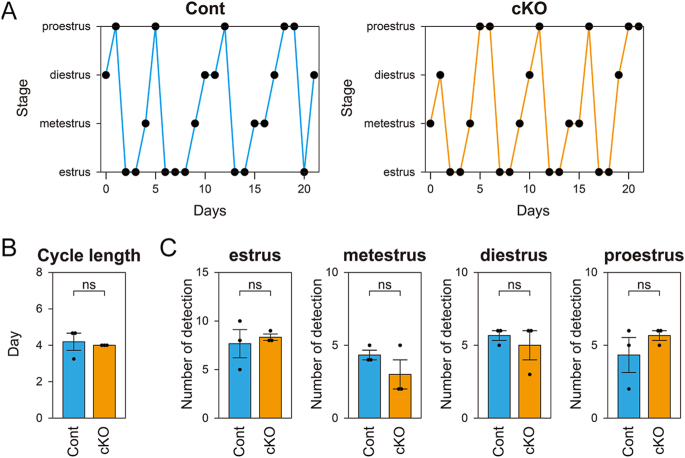
Estrous cycle analysis in control (Cont) and *Fgfr2* cKO (cKO) females. (A) Representative estrous cycle patterns in control and *Fgfr2* cKO mice. (B) Length of the estrous cycle in control and *Fgfr2* cKO mice (*n* = 3 per genotype), calculated as the interval between two consecutive estrus phases over a 3-week test period. (C) Numbers of each phase of the estrous cycle (estrus, metestrus, diestrus, and proestrus) detected in control and *Fgfr2* cKO mice (*n* = 3 per genotype) over the 3-week period. No statistically significant differences were observed between genotypes. ns, not significant (*P* > 0.05, Student’s *t*-test).

To assess the effect of FGFR2 signaling disruption on the estrous cycle, vaginal cytology was analyzed over a 3-week period (Fig. 4). Both control and *Fgfr2* cKO mice exhibited a regular estrous cycle (Fig. 4A) with an average length of approximately 4 days (Fig. 4B). In addition, no significant differences were observed in the numbers of each phase of the estrous cycle detected over the 3-week period (Fig. 4C). Therefore, loss of FGFR2 function in granulosa cells does not affect the estrous cycle.

### Cumulus expansion in *Fgfr2* cKO mice

During ovulation, cumulus cells, which are a subpopulation of granulosa cells surrounding the oocyte, produce a hyaluronan-rich extracellular matrix. This process of cumulus expansion is a prerequisite for normal ovulation, as evidenced by the defective ovulation phenotypes observed in mutant mice deficient in this function (Davis *et al.* 1999, Varani *et al.* 2002, Fulop *et al.* 2003). Since several *in vitro* studies have demonstrated that FGFs influence cumulus expansion in mammals (see Discussion for details), we next assessed whether cumulus expansion is impaired in *Fgfr2* cKO mice.

**Figure 5:**
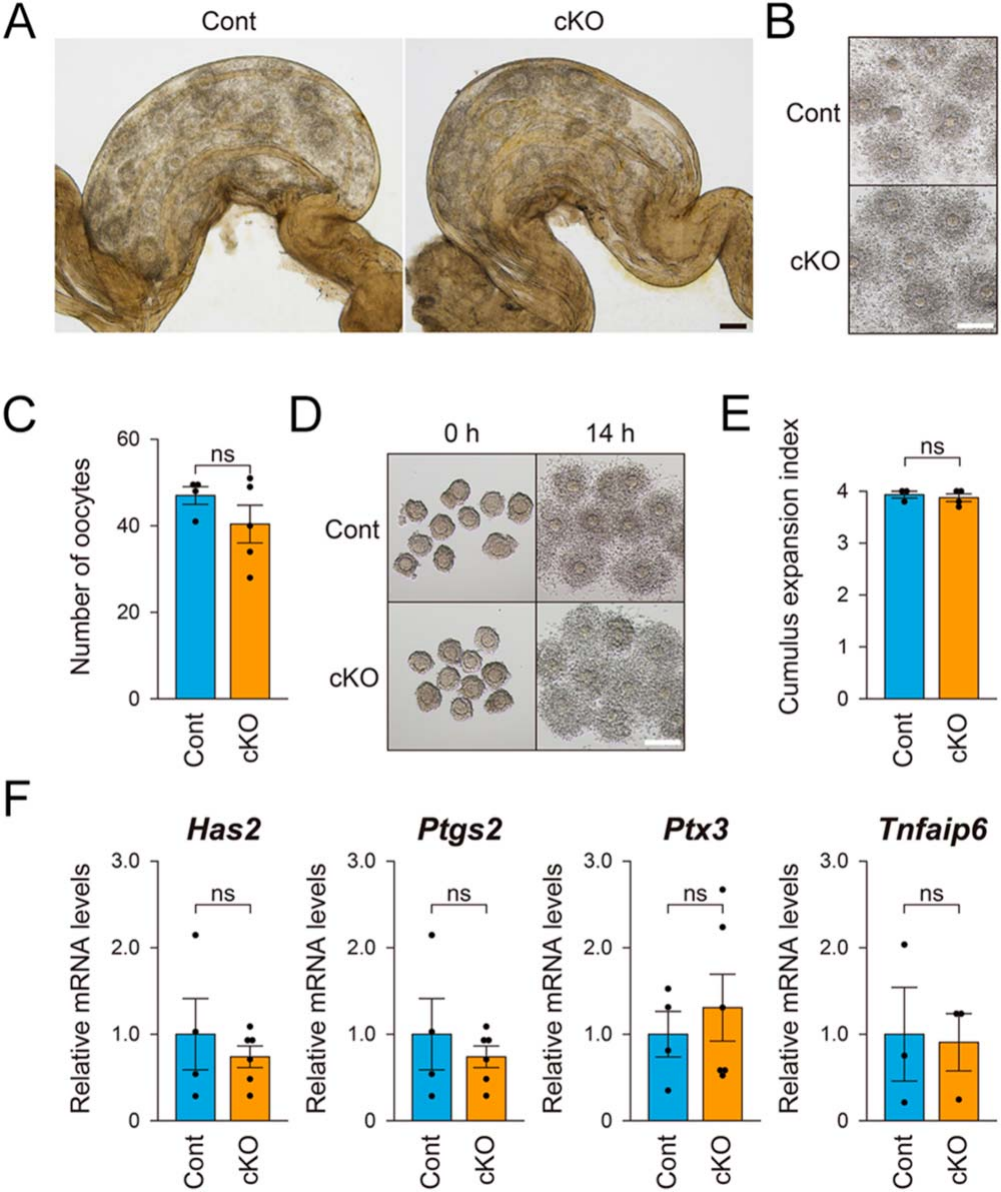
Assessment of cumulus expansion competence in COCs from control (Cont) and *Fgfr2* cKO (cKO) females. (A) Representative photographs of oviductal ampullae from control and *Fgfr2* cKO females after superovulation. Both groups exhibited well-expanded COCs in the oviducts. (B) Representative photographs of COCs extracted from the ampullae shown in (A). Scale bar = 200 μm. (C) Ovulation was induced in control (*n* = 4) and *Fgfr2* cKO (*n* = 5) mice, and the ovulated oocytes in the oviductal ampullae were counted. (D) Representative photographs of COCs before (0 h) and after (14 h) EGF stimulation *in vitro*. Scale bar = 100 μm. (E) Cumulus expansion index and (F) transcript levels of cumulus expansion-related transcripts were assessed after 14 and 4 h of EGF treatment, respectively. No statistically significant differences were observed between *Fgfr2* cKO and control mice. ns, not significant (*P* > 0.05, Student’s *t*-test).

Ovulation was induced by superovulation treatment, and the degree of cumulus expansion was evaluated both within the oviduct and after extraction from it (Fig. 5A and B). No obvious differences were observed between the expanded COCs of the cKO and control mice, suggesting normal cumulus expansion in the cKO mice. Moreover, the numbers of ovulated oocytes were comparable between cKO and control mice (Fig. 5C).

Physiologically, cumulus expansion is induced by the luteinizing hormone surge and is mediated by EGF-like peptides (Park *et al.* 2004). Therefore, this process can be mimicked *in vitro* by supplementing the culture medium with EGF. Using EGF-stimulated cumulus expansion as a model, we next assessed the ability of COCs to undergo expansion in greater detail. COCs isolated from cKO or control mice were stimulated with EGF, and the degree of expansion, along with the levels of transcripts encoding proteins essential for this process, were examined (Fig. 5D). EGF stimulation promoted cumulus expansion in COCs from both cKO and control mice. The degree of expansion, as measured by the cumulus expansion index (Eppig *et al.* 1993), was comparable between the two groups (Fig. 5E). Expression levels of the cumulus expansion-related transcripts also did not differ significantly (Fig. 5F). These findings suggest that cumulus expansion and the capacity of cumulus cells to execute this process are independent of FGFR2 signaling.

### Metabolic cooperativity between oocytes and cumulus cells in *Fgfr2* cKO mice

**Figure 6:**
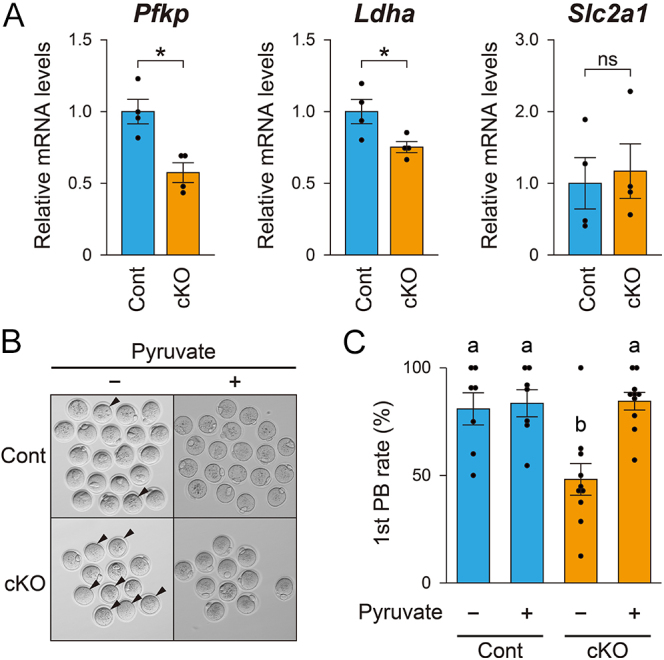
Assessment of cumulus cell competence to support oocyte meiotic resumption *in vitro* in control (Cont) and *Fgfr2* cKO (cKO) female mice. (A) Transcript levels of the glycolytic enzymes PFKP and LDHA, and the glucose transporter SLC2A1, in cumulus cells from control and *Fgfr2* cKO mice, measured by real-time PCR. *P < 0.05 (Student’s *t*-test). (B) Representative photographs of oocytes after *in vitro* maturation in the presence or absence of pyruvate. Maturation was performed using COCs; after 14 h of culture, cumulus cells were removed, and the first polar body (1st PB) was assessed. Arrowheads indicate oocytes that failed to extrude the first polar body. (C) Percentage of oocytes that extruded the first polar body. Different letters (a and b) indicate significant differences (*P* < 0.05, Tukey–Kramer test).

Oocytes are deficient in performing glycolysis and therefore cannot efficiently utilize glucose as an energy substrate (Biggers *et al.* 1967). In contrast, cumulus cells possess high glycolytic activity and actively metabolize glucose, providing the oocyte with glycolytic metabolites such as pyruvate (Donahue & Stern 1968, Leese & Barton 1985). Our previous studies showed that oocyte-derived FGF8, in combination with BMP15, promotes glycolysis in cumulus cells by enhancing expression of glycolytic enzymes, including PFKP and LDHA, *in vitro* (Sugiura *et al.* 2005, Sugiura *et al.* 2007). Moreover, FGF signaling has been suggested to promote glucose transporter expression in bovine cumulus cells (Caixeta *et al.* 2013). Therefore, the expression levels of transcripts encoding PFKP and LDHA, as well as glucose transporters (SLC2A1 and SLC2A4, also known as GLUT1 and GLUT4, respectively), in COCs were compared between cKO and littermate control mice. As shown in Fig. 6A, both *Pfkp* and *Ldha* transcripts were significantly lower in cKO mice compared with controls. In contrast, *Slc2a1* expression was not significantly altered, whereas *Slc2a4* was barely detectable, consistent with a previous report indicating that *Slc2a4* is undetectable in mouse ovaries (Zhou *et al.* 2000).

Metabolic support from cumulus cells is required for the resumption of meiotic maturation when oocytes are cultured in a medium containing glucose as the sole energy source. However, this requirement can be bypassed by supplementing the medium with glycolytic products such as pyruvate (Biggers *et al.* 1967). Using this *in vitro* model, we tested whether *Fgfr2*-deficient cumulus cells were competent to support oocyte metabolism. As shown in Fig. 6B and C, while most control oocytes resumed meiosis and extruded the first polar body regardless of pyruvate supplementation, the proportion of cKO oocytes doing so was significantly reduced in pyruvate-free medium. This reduction was rescued by pyruvate supplementation (Fig. 6C). Therefore, the cumulus cells of cKO mice were not as competent as control cumulus cells in supporting oocyte energy metabolism, at least *in vitro*.

### Developmental competence of *Fgfr2*-cKO mouse oocytes

**Figure 7:**
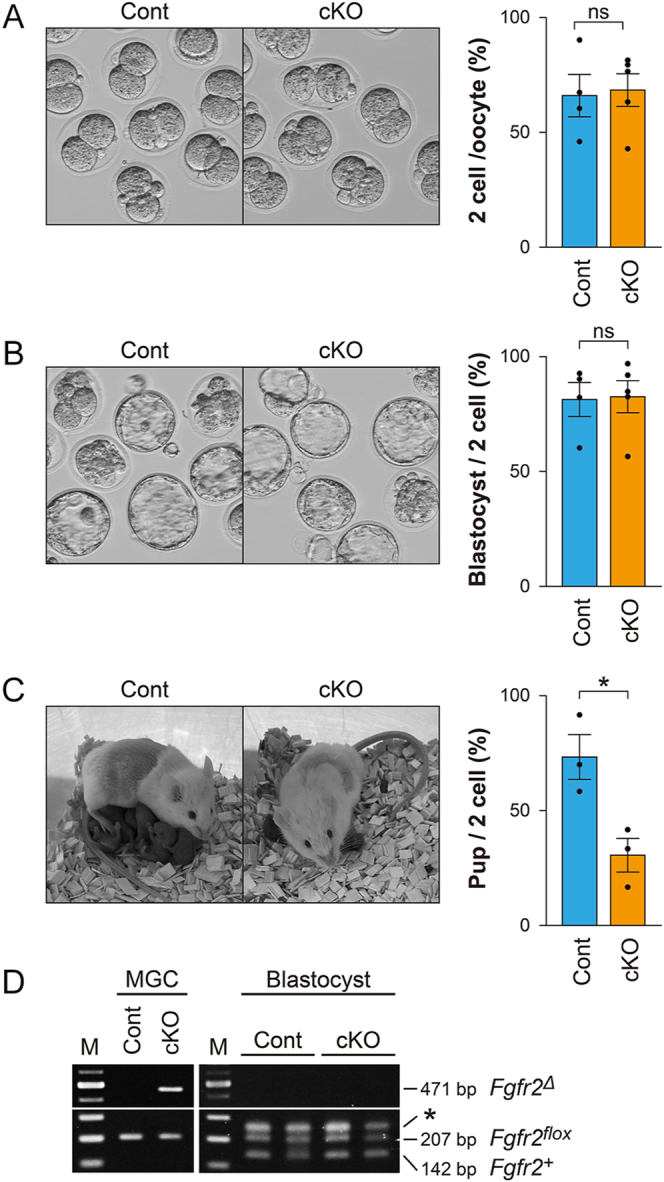
Assessment of oocyte developmental competence in control (Cont) and *Fgfr2* cKO (cKO) female mice. Oocytes from control and *Fgfr2* cKO females were fertilized *in vitro*, and preimplantation development was assessed at (A) 24 h and (B) 96 h post-IVF. (A) Left: representative photographs of 2-cell embryos 24 h post-IVF. Right: percentage of oocytes/embryos that developed to the 2-cell stage. (B) Left: representative photographs of embryos 96 h post-IVF. Right: percentage of 2-cell embryos that developed to the blastocyst stage. No statistically significant differences were observed between *Fgfr2* cKO and control groups. ns, not significant (*P* > 0.05, Student’s *t*-test). (C) Left: representative photograph of a foster mother with pups derived from transferred embryos. Right: percentage of transferred 2-cell embryos that developed into live pups. *P < 0.05 (Student’s *t*-test). (D) Detection of *Fgfr2* alleles in mural granulosa cells (MGC) and blastocysts following IVF of cKO or control oocytes. Two blastocysts were used to prepare each sample. A nonspecific amplification product of approximately 250 bp was detected in the blastocyst samples (denoted with an asterisk).

We next examined whether the developmental competence of oocytes was compromised in *Fgfr2* cKO mice. Oocytes from cKO and control mice were fertilized *in vitro*, and their preimplantation development to the blastocyst stage was assessed (Fig. 7A and B). The percentages of oocytes reaching the 2-cell stage and of 2-cell embryos reaching the blastocyst stage were not significantly different between cKO and control groups (Fig. 7A and B). However, when 2-cell embryos were transferred to wild-type females, the birth rate was significantly lower in the cKO group (30.6 ± 7.3%) than in controls (73.3 ± 9.8%) (*P* < 0.05) (Fig. 7C). Therefore, although *Fgfr2* cKO oocytes can successfully progress to the blastocyst stage *in vitro*, their competence to support full-term development is reduced.

Since the *Amhr2^Cre^* allele has been reported to be expressed at low levels in oocytes (Jorgez *et al.* 2004), we next examined whether Cre-mediated deletion of the *Fgfr2* gene occurs in cKO oocytes. To test this, oocytes from either cKO or control mice were fertilized with wild-type sperm, and the presence of the recombined *Fgfr2* allele (i.e. *Fgfr2^Δ^*) was assessed in blastocyst-stage embryos. As shown in Fig. 7D, both the floxed allele (from oocytes) and the wild-type allele (from sperm) were detected in blastocysts derived from both cKO and control mice. However, while the *Fgfr2^Δ^* allele was detected in mural granulosa cells of cKO mice, it was absent in control mural granulosa cells and in embryos. These findings strongly suggest that the reduced developmental competence of cKO mice is not attributable to *Fgfr2* deletion in oocytes or their derived embryos.

## Discussion

While the essential role of FGF signaling in follicular development has been demonstrated *in vitro*, direct *in vivo* evidence remains limited. Here, we show that female mice lacking FGFR2 signaling in granulosa cells are subfertile. Although follicular development, cumulus expansion, ovulation, and the estrous cycle appeared normal, the developmental competence of oocytes was significantly reduced. Transcript levels of the glycolytic enzymes PFKP and LDHA were significantly reduced in cumulus cells from *Fgfr2* cKO mice. This reduction likely impaired the metabolic support to oocytes, as reflected by the decreased ability of cKO cumulus cells to promote meiotic resumption *in vitro*. These findings are consistent with our previous reports showing that oocyte-derived FGFs, in coordination with BMP15, promote glycolysis in cumulus cells *in vitro* (Sugiura *et al.* 2005, Sugiura *et al.* 2007). Given the importance of metabolic support from cumulus cells for oocyte development (Turathum *et al.* 2021), our data suggest that metabolic insufficiency in cumulus cells contributes, at least in part, to the reduced developmental competence of oocytes in *Fgfr2* cKO mice. This study is the first to demonstrate an essential *in vivo* role for follicular FGF signaling in regulating cumulus cell glycolysis and supporting female fertility in mammals.

In this study, we employed *Fgfr2* cKO mice to investigate the functional roles of FGF signaling in follicular development. However, it is noteworthy that granulosa cells express *Fgfr1* and *Fgfr4* in addition to *Fgfr2* mRNA. Therefore, the *Fgfr2* cKO model does not represent a complete ablation of follicular FGF signaling. Nevertheless, we observed a significant reduction in *Fgfr1* mRNA levels in addition to the expected reduction in *Fgfr2* mRNA and a downward trend in *Fgfr4* mRNA expression in granulosa cells of *Fgfr2* cKO mice. While the precise mechanism by which *Fgfr2* deficiency affects *Fgfr1* and *Fgfr4* mRNA expression remains unclear, a positive correlation between *FGFR1* and *FGFR2* expression has been documented in human laryngeal squamous cell carcinoma tissues (Hu & Zhang 2022). These observations suggest the potential existence of a feedback regulatory mechanism in which FGF signaling modulates the expression of individual FGFRs, implying that the overall FGF signaling cascade may be perturbed in granulosa cells of *Fgfr2* cKO mice. Given the reduction in multiple *Fgfr* mRNA transcripts, the present *Fgfr2* cKO model may represent a state of compromised FGF signaling in granulosa cells.

*Fgfr1–3* undergo alternative splicing within the third immunoglobulin-like loop, giving rise to the IIIb and IIIc isoforms, which exhibit markedly different binding specificities for FGF ligands (Ornitz *et al.* 1996, Zhang *et al.* 2006). Distinct ovarian expression patterns of these isoforms have been reported, primarily in cattle (Buratini *et al.* 2005, Price 2016, Schutz *et al.* 2022), and their divergent ligand specificities suggest that they may fulfill non-overlapping roles in follicular development. Expression also appears to be species-specific: in cattle, *Fgfr3c* is expressed in granulosa cells, whereas in mice *Fgfr3* expression, regardless of isoform, has not been detected in granulosa cells (present study) or in pregnant ovaries (Puscheck *et al.* 1997). Both the IIIb and IIIc isoforms of *Fgfr2* have been identified in bovine granulosa cells (Berisha *et al.* 2004, Zhang & Ealy 2012). The floxed *Fgfr2* allele employed in the present study deletes both isoforms; consequently, the specific contributions of individual isoforms could not be determined. Future elucidation of isoform-specific roles of *Fgfr2* using conditional knockout models targeting individual isoforms will be essential for defining their distinct physiological functions and clarifying their contributions to ovarian development and fertility.

The *Amhr2^cre^* knock-in mouse strain has been widely used to recombine floxed alleles, including *Rb*, *Smads*, and *paxillin*, in granulosa cells (Andreu-Vieyra *et al.* 2008, Li *et al.* 2008, Pangas *et al.* 2008, Vann *et al.* 2023). Expression of the *Amhr2^cre^* allele in ovaries has been reported to occur primarily in granulosa cells of growing follicles, but not in primordial or primary follicles (Jeyasuria *et al.* 2004, Jorgez *et al.* 2004). Nevertheless, some studies have also detected Cre activity in the ovarian surface epithelium (Fan *et al.* 2009) and low-level expression in theca cells and oocytes (Jorgez *et al.* 2004). In the present study, however, no recombined alleles were detected in embryos obtained after IVF of cKO oocytes, indicating that the reduced developmental competence of cKO oocytes is unlikely to result from *Fgfr2* deletion in oocytes.

Although meiotic resumption of cKO mouse oocytes was suppressed under culture conditions lacking pyruvate, meiotic resumption *in vivo* did not appear to be affected. Indeed, no immature (i.e., germinal vesicle-stage) oocytes were observed among ovulated oocytes after superovulation in cKO mice (data not shown). One possible explanation for this discrepancy between the *in vivo* and *in vitro* observations is that other intrafollicular FGF signaling pathways may compensate for the reduction in FGFR2 signaling, thereby providing sufficient energy support for meiotic resumption of oocytes *in vivo*. However, the present results demonstrated that transcripts encoding glycolytic enzymes were downregulated, suggesting that no other signaling pathways compensate for the reduction in FGFR2 to maintain their expression in cKO mice. Another possibility is that, *in vivo*, unlike *in vitro* conditions where pyruvate is supplied only by cumulus cells, pyruvate may be delivered from the bloodstream or surrounding tissues. Therefore, the reduced fertility observed in cKO mice is unlikely to result from impaired meiotic resumption but rather from insufficient oocyte development due to sustained metabolic insufficiency in cumulus cells throughout oocyte growth.

Oocytes from *Fgfr2* cKO mice developed normally to the blastocyst stage *in vitro*, yet their ability to develop to term after embryo transfer was significantly reduced. This suggests that defects beyond the preimplantation embryonic stage contribute to the subfertility phenotype. The mechanism by which reduced metabolic support from cumulus cells during oocyte development impairs post-implantation competence remains unclear and requires further investigation. However, because alterations in pyruvate metabolism modulate histone acetylation status in oocytes (Alcantara da Silva *et al.* 2024), and because histone acetylation critically influences oocyte developmental competence (Akiyama *et al.* 2006), it is plausible that impaired acetylation contributes to this phenotype. Indeed, previous studies have shown that treatment with trichostatin A, a histone deacetylase inhibitor, induces histone hyperacetylation and increases the incidence of spindle defects in mouse and porcine oocytes (De La Fuente *et al.* 2004, Wang *et al.* 2006). Given that aneuploidy resulting from chromosome segregation errors can cause post-implantation pregnancy loss, disrupted histone acetylation may contribute to the reduced fertility observed in *Fgfr2* cKO mice. Therefore, further research is warranted to examine histone acetylation status in cKO mice and determine whether its disruption contributes to the reduced developmental competence of their oocytes.

Cumulus expansion was not affected in *Fgfr2* cKO mice. Several studies have found that FGF signaling can exert either positive or negative effects on cumulus expansion, depending on the species and experimental conditions. For example, bovine oocytes secrete FGF10 and FGF17 (Buratini *et al.* 2007, Machado *et al.* 2009), which promote cumulus expansion *in vitro* (Caixeta *et al.* 2013, Machado *et al.* 2015), whereas FGF2 appears to negatively affect this process (Barros *et al.* 2019). In mice, FGF2 has been shown to promote cumulus expansion *in vitro* (Du *et al.* 2021), whereas our previous report suggested that some FGFs suppress it (Kanke *et al.* 2022). The normal cumulus expansion observed in the *Fgfr2* cKO mice in the present study suggests that FGFR2 signaling may be dispensable for this process or that compensatory mechanisms involving other FGFRs maintain normal cumulus expansion *in vivo*. Further studies are needed to clarify the specific role of FGF signaling in cumulus expansion and to determine whether other pathways can compensate for the loss of FGFR2.

Reaggregated follicles with developmental stage mismatches between oocytes and somatic cells develop in alignment with the oocyte stage (Eppig *et al.* 2002). This strongly suggests that oocytes play a central role in determining the rate of follicular development. The precise mechanisms by which oocytes control follicular development remain unclear; however, it is hypothesized that oocytes contribute, at least in part, by regulating granulosa cell metabolism, including glycolysis (Su *et al.* 2009, Sugiura *et al.* 2023). Therefore, we expected that ovaries from *Fgfr2* cKO mice would exhibit delayed follicular development. Interestingly, however, follicular development was histologically normal in the cKO mice, despite likely reductions in glycolytic activity in cKO cumulus cells. Further studies are needed to clarify the extent to which glycolysis contributes to oocyte regulation of follicular development and whether additional oocyte-controlled metabolic pathways, such as cholesterol biosynthesis (Su *et al.* 2008), are also involved.

In addition, several studies, primarily using bovine models, have demonstrated that FGF signaling plays an essential role in the development and function of the corpus luteum, most likely through the regulation of angiogenesis (Min *et al.* 2024, Ziemak *et al.* 2025). In the present study, although not examined in detail, no obvious abnormalities were observed in the number or morphology of the corpora lutea in cKO mice, at least within the scope of ovarian histological assessment. Further investigations are warranted to clarify the role and involvement of FGFs and FGFR2 in corpus luteum function in mice.

Our findings provide new insight into the role of FGF signaling in ovarian function and female fertility. Although the mechanisms linking FGF signaling to oocyte competence remain to be fully elucidated, our results indicate that FGF signaling in granulosa cells is essential for maintaining the metabolic environment necessary for oocyte development. Future studies should explore whether restoring metabolic function in cumulus cells can rescue the developmental defects observed in *Fgfr2* cKO mice. In addition, investigating potential epigenetic alterations in oocytes may yield further insight into how FGF signaling influences long-term reproductive outcomes.

## Declaration of interest

KS is an Associate Editor of *Reproduction* and was not involved in the editorial process of this manuscript. The authors declare that there is no conflict of interest that could be perceived as prejudicing the impartiality of the research reported.

## Funding

This work was supported by a Grant-in-Aid for Exploratory Research from the Japan Society for the Promotion of Sciencehttps://doi.org/10.13039/501100001691 (Nos. 23K27052 to KS).

## Author contribution statement

TK, AI, YA, and SM performed the experiments. TK, AI, YA, SM, WF, TE, KN, and KS analyzed the data. WF, TE, KN, and KS designed the experiments. TK and KS wrote the paper. All authors have read and approved the final manuscript.
